# Lung Cancer Screening with Low-Dose CT: Radiation Risk and Benefit–Risk Assessment for Different Screening Scenarios

**DOI:** 10.3390/diagnostics12020364

**Published:** 2022-02-01

**Authors:** Elke A. Nekolla, Gunnar Brix, Jürgen Griebel

**Affiliations:** Department of Medical and Occupational Radiation Protection, Federal Office for Radiation Protection, Ingolstaedter Landstrasse 1, 85764 Oberschleissheim, Germany; gbrix@bfs.de (G.B.); juergen-griebel@gmx.de (J.G.)

**Keywords:** lung cancer screening, low-dose CT, radiation risks, radio-epidemiological models, benefit–risk ratios

## Abstract

Lung cancer is a severe disease that affects predominantly smokers and represents a leading cause of cancer death in Europe. Recent meta-analyses of randomized controlled trials (RCTs) have yielded that low-dose computed tomography (LDCT) screening can significantly reduce lung cancer mortality in heavy smokers or ex-smokers by about 20% compared to a control group of persons who did not receive LDCT. This benefit must be weighed against adverse health effects associated with LDCT lung screening, in particular radiation risks. For this purpose, representative organ doses were determined for a volume CT dose index of 1 mGy that can be achieved on modern devices. Using these values, radiation risks were estimated for different screening scenarios by means of sex-, organ-, and age-dependent radio-epidemiologic models. In particular, the approach was adjusted to a Western European population. For an annual LDCT screening of (ex-)smokers aged between 50 and 75 years, the estimated radiation-related lifetime attributable risk to develop cancer is below 0.25% for women and about 0.1% for men. Assuming a mortality reduction of about 20% and taking only radiation risks into account, this screening scenario results in a benefit–risk ratio of about 10 for women and about 25 for men. These benefit–risk ratio estimates are based on the results of RCTs of the highest evidence level. To ensure that the benefit outweighs the radiation risk even in standard healthcare, strict conditions and requirements must be established for the entire screening process to achieve a quality level at least as high as that of the considered RCTs.

## 1. Introduction

Lung cancer is a severe disease that predominantly affects heavy smokers or ex-smokers and represents with an estimated 1.8 million cases worldwide the leading cause of cancer death in 2020 [1]. If lung cancer is detected at an early stage, the success of therapy can be significantly improved, so that early detection plays an important role.

Due to technological progress in recent years, low-dose computed tomography (LDCT) offers a promising perspective for the early detection of lung cancer in asymptomatic (ex-)smokers. A meta-analysis of randomized controlled trials (RCTs) recently presented by our group [2] underlines that LDCT screening can significantly reduce lung cancer mortality in heavy (ex-)smokers by about 20% compared to a non-screened group. However, this benefit must be carefully weighed against adverse health effects associated with this screening approach.

This applies in particular for radiation risks unavoidably related to CT screening examinations of the chest. In this context, it is essential to consider that only a small portion of screening participants will benefit from the examinations due to the low prevalence of lung cancer in the target population, while all participants will be subjected to the risks associated with the test [3]. The European Directive 2013/59/Euratom [4] underlines this dilemma and thus requires a specific justification for each medical radiological procedure performed on asymptomatic individuals for early detection of disease (article 55 para. 2 letters f, h).

It is the aim of the present article (i) to give an overview of current radiation risk models with respect to lung cancer LDCT screening, including some that consider possible interactions between radiation and smoking, and (ii) to provide conservative estimates, for different screening scenarios, addressing both lifetime radiation risks and benefit–risk ratios associated with the LDCT examinations. The focus of this study is to obtain appropriate and up-to-date estimates of radiation risk (vs. benefit) for a Western European population using current German baseline data. Due to the relatively low radiation exposure of LDCT, only stochastic radiation effects are relevant in this context and thus only these will be dealt with. This especially includes the radiation-induced development of malignant diseases that can occur years or even decades after radiation exposure (latency period). Since various aspects of analyzing and modelling stochastic radiation risks are still subject of scientific debate, radiation risks in this study will be estimated using conservative assumptions that are consistent with radio-epidemiological data obtained over many decades.

## 2. Theoretical Background

### 2.1. Basic Assumptions and Study Cohorts of Persons Exposed to Ionizing Radiation

The risk of radiation-induced cancers and leukaemias is derived from radio-epidemiological studies of large cohorts of persons who were exposed to ionizing radiation and appropriate control groups of non-exposed or low-exposed persons. Data on the type and level of radiation exposure, age at exposure, age attained during follow-up, gender as well as type and time of disease and/or disease-specific deaths occurring during observation are recorded over a long period of time. The cancer rates observed in the exposed cohort are compared with those of the control group to derive dose-dependent risk estimates. In addition, other parameters that may influence radiation risk, such as age and gender, can also be accounted for in these risk analyses (e.g., [5]). 

By means of radio-epidemiological studies, however, significant increases in cancer risk can only be detected at dose levels that are much higher than the radiation doses associated with most X-ray examinations. Even for a large cohort of persons exposed to low doses of only a few millisieverts (mSv), stochastic radiation effects cannot be observed with statistical significance [6]. For the low dose range, therefore, no reliable information on the dose–effect relation is available, so that one has to rely on an extrapolation of the effects observed in the higher dose range towards lower doses. For this purpose, radiation protection assumes a proportional relationship between cancer risk and dose without assuming a threshold dose (linear-non-threshold, LNT hypothesis). According to the International Commission on Radiological Protection (ICRP), the use of the LNT model represents “the best practical approach to managing risk from radiation exposure and commensurate with the ‘precautionary principle’” [7]. Furthermore, for solid cancers, the ICRP assumes a dose and dose rate effectiveness factor (DDREF) of 2 for the low dose range. In fact, the DDREF is a reduction factor that is intended to account for the lower biological effectiveness (per dose unit) of radiation exposure assumed by the ICRP at low doses and low dose rates compared with an exposure at high doses and high dose rates. Other commissions adopt a DDREF of 1.5 (BEIR VII Committee [5]) or of 1 (German Radiation Protection Commission, SSK [8]). 

The assessment of radiation risks at higher doses can rely on a solid database of epidemiological studies. The so-called Life Span Study (LSS) of survivors of the atomic bombings in Hiroshima and Nagasaki exposed to acute gamma (and to some extent neutron) radiation is the main source of current knowledge on radiation risks due to its size (more than 85,000 persons of both sexes and all ages with dose estimates), the long observation period (>50 years) and the large internal control group of low- or non-exposed survivors. It is thus the most important basis for quantitative risk assessments [5,9]. For the Japanese LSS, data are available on both cancer incidence (follow-up from 1958, the start of cancer registries in Hiroshima and Nagasaki) and cancer mortality (follow-up from 1950). In addition to the Japanese LSS, there are also numerous study cohorts of persons who were exposed to X-ray or other radiation for diagnostic or therapeutic reasons, e.g., the study by Boice et al. [10] in which women received pneumothorax therapy for tuberculosis and had repeated chest X-rays for control. As with breast cancer, the evidence on radiation risk in lung cancer has been derived not only from the Japanese LSS but also from studies of patient populations undergoing radiotherapy for malignant or benign diseases, such as Hodgkin’s lymphoma [11] or ankylosing spondylitis [12]. For an overview of relevant studies, see [5] or [13].

### 2.2. Risk Model Approaches: Absolute and Relative Risk Models

In the following, the terminology primarily refers to cancer incidence. But the terms introduced for this purpose apply accordingly to cancer mortality. For reasons of simplicity, a separate parameter for gender is omitted.

The age-specific baseline (or background) rate *r*_0_(*a*) is the rate of a specific type of cancer occurring in a normal population at a given age, *a*. The excess absolute rate, *ear*(*a*, *e*, *D*), is the age-specific absolute rate of cancer caused by radiation exposure at a certain age, *e*, to an organ equivalent dose, *D*. In one approach, the radiation-related increased cancer rate, *r*(*a*, *e*, *D*), is given by the sum:

(1) $$r\left(a,e,D\right)=r_{0}\left(a\right)+ear\left(a,e,D\right)$$

In this so-called absolute (or additive) risk model it is assumed that the excess absolute rate is independent of the background rate. An alternative approach, the so-called relative (or multiplicative) risk model, is based on the assumption that the excess rate depends on the background rate, i.e., is a certain multiple of the background rate:

(2) $$r\left(a,e,D\right)=r_{0}\left(a\right)\cdot \left(1+err\left(a,e,D\right)\right)$$

with $err\left(a,e,D\right)=ear\left(a,e,D\right)/r_{0}\left(a\right)$ the so-called excess relative rate, i.e., the age-specific relative cancer rate caused by radiation exposure at age *e* to an organ equivalent dose, *D*. For example, an excess relative rate of 1 means that the cancer rate observed in the exposure group is twice as high as expected (*r* = 2 *r*_0_), i.e., compared to the baseline rate. 

Several measures of lifetime risk have been used to express radiation risks [13,14,15,16]. The BEIR VII committee has chosen to use what Kellerer et al. refer to as the lifetime attributable risk, *LAR* [16]. Denoting the minimum latency time to clinical manifestation of radiation-induced cancer by *lag, LAR* is the radiation-related excess risk of developing cancer from *e* + *lag* to the end of life, *a*_max_. Correspondingly, the *LAR* is the excess absolute cancer rate, *ear*, integrated over time from *e* + *lag*. To account for competing (life-shortening) risks, *ear* is usually multiplied by the age-specific conditional survival probability *S*(*a*, *e*) of a person who has already reached age *e* [5]:

(3) $$LAR\left(e,D\right)=\int_{e+lag}^{a_{\max}}\;S\left(t,e\right)\cdot ear\left(t,e,D\right)\;dt\;$$

### 2.3. Risk Transfer between Populations with Different Cancer Rates

For gamma radiation, and thus also for X-rays, risk estimates or risk models are mostly based on analyses of data of the LSS of Japanese atomic bomb survivors. When transferring risk estimates to Western populations, the question arises whether the relative risk model (multiplicative transfer model) can be used to determine the excess risk in the Western population, or whether the absolute risk model (additive transfer model) is more appropriate. In the first case, in Equation (2) the baseline rate of the Western population is to be used, while in the second case only the excess rate of the exposed (Japanese) study cohort is accounted for (Equation (1)). The same problem arises if the normal rates of two cohorts differ due to different non-radiation risk factors, e.g., due to strongly differing smoking behavior. 

**Figure 1:**
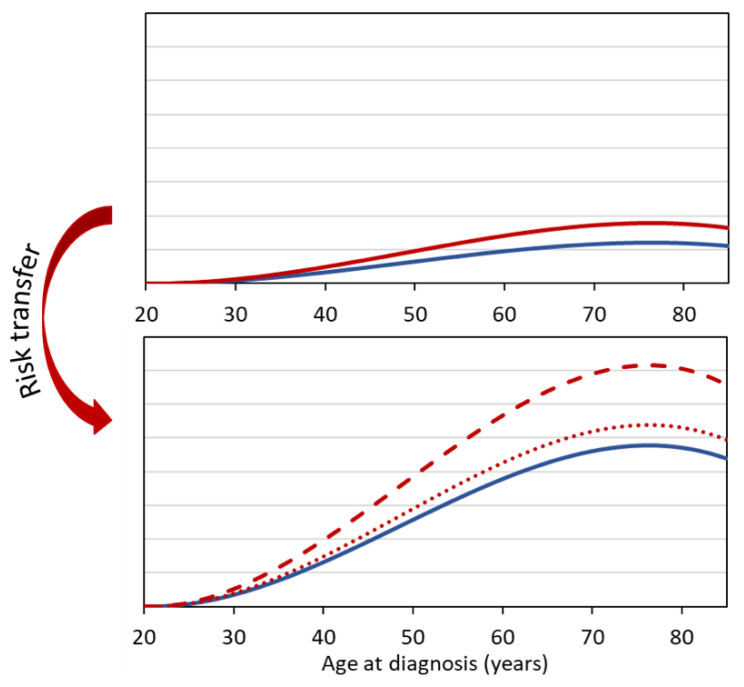
Risk transfer from an exposed population with low baseline rates (blue line in upper panel) to a population with high baseline rates (blue line in lower panel). The scaling of the two graphs is the same. In the upper chart, the red line shows the radiation-related increased rate (*err* = 1.5, i.e., 50% increase). A risk transfer using the multiplicative model results in the red dashed line, while a risk transfer using the additive model results in the red dotted line.

For cancers for which baseline rates vary substantially between different populations, the method of risk transfer is crucial. For example, the baseline rates for breast cancer in Germany are considerably higher than the breast cancer rates in Japan. Similarly, lung cancer rates are significantly higher in heavy smokers than in moderate or non-smokers. Figure 1 illustrates that in such cases the choice of the transfer model (multiplicative or additive approach) leads to significantly different results for the estimated radiation risk.

Besides the purely additive or multiplicative model, there are also more complex models such as the models of the BEIR VII Committee [5] or models that consider a possible interaction between radiation and smoking [17].

### 2.4. The Risk Models of the BEIR VII Committee

In 2006, the most recent report of the BEIR Committee was published [5]. It is the seventh report in a series of contributions of the National Research Council of the USA, dealing with the effects of ionizing radiation in the low dose range. On the one hand, the BEIR VII report is a comprehensive review of the most recent radiation biological, biophysical, and radiation epidemiological literature at the time of evaluation. On the other hand, the report also gives comprehensive radiation risk models for cancer incidence and mortality. These are based on data from the LSS of Japanese atomic bomb survivors as well as on meta-analyses of data from persons exposed for diagnostic or therapeutic reasons. 

The BEIR VII Committee considered both relative and absolute risk models to model the radiation-related excess rates defined in Equations (1) and (2). The models depend not only on organ equivalent dose, *D*, and gender, but also on age attained, *a*, and, in the case of leukemia, time since exposure, *t*. The risk models include linear dose response relationships for all solid tumors and a linear quadratic dose relationship for leukemias. Again, a separate parameter to account for the influence of gender is omitted.

For persons exposed after age 30, the models are:

• For solid malignant tumors (except breast cancer and thyroid cancer)

(4) err(a,D)ear(a,D)}=β ·D ·(a60)η

• For leukemias

(5) err(t,D)ear(t,D)}=β·D·(1+θ·D)·(t25)α

The parameters denoted by Greek letters are the result of maximum likelihood analyses to the Japanese LSS data. These parameters differ for the different organs and for the relative and absolute risk models. The parameter β differs also for male and female persons. 

For breast and thyroid cancer, the risk models used by the BEIR VII Committee deviate from those in Equations (4) and (5). For thyroid cancer, no absolute risk model was used, but only a relative risk model with an age at exposure dependence based on a meta-analysis by Ron et al. [18]:

(6) err(e,D)=β·D·exp(γ·(e−30))

This meta-analysis demonstrated that the excess risk was significantly affected by age at exposure, *e*, with a strong decrease in risk with increasing age at exposure. 

For breast cancer, an absolute risk model with dependencies on age at exposure and attained age based on a meta-analysis by Preston et al. [19] was considered:

(7) ear(a,e,D)=β·D·exp(γ·(e−30))·(a60)η

In the study by Preston et al., a pronounced dependence of the excess absolute risk per dose was observed both on age at exposure and on age attained, with a decrease with age of exposure and an increase with attained age. For the Japanese LSS data on breast cancer, the BEIR VII Committee also performed risk modeling for a relative risk model. However, because Preston et al. [19] also included data from Western cohorts in their modeling, the BEIR VII Committee favored the absolute risk model (Equation (7)).

With the exception of thyroid and breast cancer, a mixed risk transfer approach was used for other cancers. Accounting for both models, the *LAR* (Equation (3)) estimates resulting from the relative risk model, *LAR*_rel_*,* and the *LAR* estimates resulting from the absolute risk model, *LAR*_abs_, were combined by calculating the geometric mean with different weights:

(8) $$LAR=LAR_{\mathrm{rel}}{}^{w_{\mathrm{rel}}}\;\cdot \;LAR_{\mathrm{abs}}{}^{w_{\mathrm{abs}}}$$

where *w*_rel_ = 0.7 and *w*_abs_ = 0.3 for cancers other than breast, thyroid and lung cancer and *w*_rel_ = 0.3 and *w*_abs_ = 0.7 for lung cancer. The BEIR VII Committee thus adopted a predominantly additive approach for lung cancer and a predominantly multiplicative approach for other cancers (except for thyroid and breast cancer). The higher weighting of the relative risk model for cancers other than breast, thyroid, and lung cancer was due to the observation that relative risk models often provide a slightly better fit to the data. In addition, the BEIR VII Committee assumed that relative risk models are less susceptible to potential bias from underreporting of cancer cases.

The BEIR VII committee estimated 95% “subjective confidence intervals (CI)” including not only random errors but also “judgmental uncertainties”. These reflect the most important sources of uncertainty, namely, statistical variation, uncertainty in the DDREF, and uncertainty in the method of risk transfer from the Japanese LSS to the U.S. population. The resulting subjective CI of the estimated *LAR* values for all solid cancers can roughly be given by the interval [0.5 *LAR*, 2 *LAR*].

### 2.5. Interaction between Radiation and Smoking for Lung Cancer Risk

The question of a possible synergistic or antagonistic interaction of the two noxious agents radiation and smoking plays an important role in the assessment of the radiation risks of lung cancer. Depending on the model, the risk of radiation-related lung cancer may be overestimated or underestimated. Four models that assume either independent or interacting risks between radiation and smoking are explained below. The additional risk caused by smoking alone is denoted by *ear*_s_(*a*), which is 0 for non-smokers.

• Simple additive model: The radiation-related excess absolute rate, *ear*(*a*, *e*, *D*), for smokers and non-smokers is equal.

(9) $$r_{s}\left(a,e,D\right)=r_{0}\left(a\right)+ear_{s}\left(a\right)+ear\left(a,e,D\right)$$

• Generalized additive model: *ear*(*a*, *e*, *D*) for smokers and for non-smokers differ.

(10) $$r_{s}\left(a,e,D\right)=r_{0}\left(a\right)+ear_{s}\left(a\right)+ear\left(a,e,D\right)\cdot \rho$$

If *ρ* is smaller than 1, the radiation-associated risk for smokers is smaller than that for non-smokers; if it is larger than 1, the radiation risk for smokers is higher than that for non-smokers.

• Simple multiplicative model: The radiation-related excess relative rate *err*(*a, e, D*) for smokers and non-smokers is equal.

(11) $$r_{s}\left(a,e,D\right)=\left(r_{0}\left(a\right)+ear_{s}\left(a\right)\right)\cdot \;\left(1+err\left(a,e,D\right)\right)$$

• Generalized multiplicative model: *err*(*e*, *a*, *D*) for smokers and for non-smokers differ.

(12) $$r_{s}\left(a,e,D\right)=\left(r_{0}\left(a\right)+ear_{s}\left(a\right)\right)\cdot \;\left(1+err\left(a,e,D\right)\cdot \rho \right)$$

with *ρ* as defined in Equation (10).

The BEIR VII Committee’s favored risk model for lung cancer gives more weight to the additive approach for the interaction between radiation and smoking than to the multiplicative approach. This was motivated by the results of a former analysis by Pierce et al. [20], which found an additive relationship between radiation and smoking. Preston et al. [21] analyzed the incidence data of solid cancers in the Japanese LSS, which was the same data set used for modeling in the BEIR VII report. Although smoking could not be explicitly accounted for in these analyses, the authors concluded that smoking and radiation may have independent (additive) effects on lung cancer risk in the Japanese LSS. In a recent study, Cahoon et al. analyzed the incidence data of the Japanese LSS (follow-up 1958–2009) for cancer in lung and other respiratory organs [22]. To characterize the combined effect of radiation and smoking, they considered both (generalized) additive and (generalized) multiplicative models. The generalized multiplicative model yields the best fit to the data. Similar to an older study of the same study population with follow-up until 1999 [17], the analyses showed a significantly higher excess relative risk per dose for lung cancer at low to moderate compared to high tobacco use. No radiation-associated excess risk was observed in heavy smokers (from one pack per day). In an analysis by Grant et al. [23], the Japanese LSS incidence data were analyzed for the sum of all solid cancers (follow-up 1958–2009). Smoking as a factor influencing the radiation-associated risk was also examined. Only the simple additive and multiplicative models were considered. The authors opted for the simple multiplicative model, although the additive model achieved a better fit of the data. However, this was for practical reasons, as it allowed comparison with previous analyses of the Japanese LSS data, where cigarette consumption was not accounted for. Overall, it was concluded that the choice of model—multiplicative or additive—had little impact on the shape of the dose–effect curve or the age dependencies of the radiation-related risk, suggesting that smoking only slightly modifies radiation risk estimates. In a study of mortality data from the Japanese LSS with follow-up until 2003 by Ozasa et al. [24], models similar to the BEIR VII models were applied, i.e., multiplicative and absolute models. No information on smoking behavior was included in the analysis. However, the radiation risk estimates are consistent with those estimated by Cahoon et al. [22] using the generalized multiplicative model for non-smokers. 

In summary, there is currently no consensus on a possible interaction of smoking and radiation. Therefore, the BEIR VII method of LAR weighting for lung cancer is used in the following estimation of the radiation risk resulting from LDCT screening.

## 3. Material and Methods

### 3.1. Risk Assessment for LDCT Screening

Prerequisite for the estimation of organ specific *LAR* is the knowledge of the organ equivalent doses, *D*, resulting from a CT screening examination that is characterized with regard to patient exposure by the respective volume CT dose index, *CTDI*_vol_, displayed on the CT system. For this purpose, the following approach was used. Organ equivalent doses were estimated for a typical LDCT examination of the chest ranging from the base of the lung to the lung apex. The calculations were performed for a variety of modern CT systems (with ≥64 detector rows) of different manufacturers that are well suited for lung cancer screening for both a female and a male mathematical body phantom by means of a validated CT dosimetry program (CT-Expo, version 2.5; Hamburg/Hannover, Germany [25,26]) taking into account the more precise anatomical organ locations in realistic voxel models. The scanner-specific organ doses were divided by the respective *CTDI*_vol_ and averaged over all CT systems considered. To obtain good approximations of organ equivalent doses for any CT scanner and protocol, the resulting representative organ-specific dose coefficients have to be multiplied by the respective *CTDI*_vol_.

Since the literature does not yet provide consistent answers to the question of the correct risk transfer, our assessment of the radiation risk of lung cancer screening is mainly based on the approach of the BEIR VII committee with the relative and absolute risk models given in Equations (4) and (5). An exception is made for the estimation of the radiation risk for breast cancer. Here, in addition to the preferred absolute risk model according to Equation (7), the relative risk model given in the BEIR VII Report was also considered. In line with a mixed approach to risk transfer, the two resulting *LAR* estimates were conservatively weighted according to Equation (8), assuming that the absolute model has more weight (*w*_rel_ *=* 0.3 and *w*_abs_ = 0.7). The higher weighting of the absolute model follows the rationale of the BEIR VII committee, which preferred the absolute model because data from Western cohorts were included in the modeling. 

For the estimation of the age-, sex-, and organ-specific *LAR* for a German population, recent German baseline rates for cancer incidence and cancer mortality [27] as well as data from German life tables for the conditional life probability were applied. Deviating from BEIR VII, a DDREF of 1 was used in our study, which yields more conservative estimates.

The differences between the BEIR VII methodology and the approach used in the present paper are summarized in Table 1.

For the *LAR* related to lung cancer screening, lung cancer rates for heavy smokers were included. This differs from our conventional approach, which uses lung cancer background rates of the German general population, i.e., rates of a population of non-smokers and (ex-)smokers with different smoking habits. Since age-specific cancer data for smokers in Germany are not available, these had to be approximated. Here it was assumed that the baseline rates for lung cancer in male and female heavy smokers are the same and that these are twice as high as the lung cancer rate of the average male population with mixed smoking habits. This hypothesis is supported by the analysis of two extensive US-American cohorts (Nurses’ Health Study with about 120,000 participants; Health Professionals Follow-up Study with about 50,000 participants) by Bain et al. [28] and the study of Cahoon et al. [22] on lung cancer risk in the Japanese LSS cohort of atomic bomb survivors.

### 3.2. Benefit–Risk Assessment of LDCT Screening

In benefit–risk assessments of screening procedures with ionizing radiation, the conventional approach is to compare the benefit (i.e., the “lives gained”) with the radiation-associated lifetime risk *LAR* (the “radiation-induced” cancer deaths). Both estimates refer to the remaining lifetime from the first screening examination on, considering a minimum latency period of 5 or 2 years for the development of a radiation-related symptomatic solid cancer or leukemia, respectably. 

A meta-analysis by Hunger et al. [2] of six RCTs on the benefit of an LDCT screening suggests a significant reduction of lung cancer mortality compared to that of a population of persons not screened. In the RCTs, included in the analysis, the intervals between screening visits were usually one year, but two studies also included intervals of 2 and 2.5 years, respectively. Between four and seven screening visits were scheduled, and the duration of follow-up since randomization was between 8 and 10 years. Studies recruited men and women between 49 and 75 years of age with a smoking history of more than 20 pack-years, i.e., (packs smoked per day) × (years as a smoker), and, for ex-smokers, less than 15 years since quitting (cf. Table 1 in [2]). The estimated relative reduction of lung cancer mortality by LDCT screening was 20% when compared to no screening (95% confidence interval: [8%,30%]). Therefore, this value was used for benefit–risk estimations. 

Another approach to benefit–risk assessment is to determine the required reduction of lung cancer mortality, *MR*, by LDCT screening to achieve at least a pre-defined benefit–risk ratio. Assuming a benefit–risk ratio of at least 10, the required benefit, *MR*_10_, is:

(13) $$MR_{10}=10\cdot LAR/LR_{0}$$

with *LR*_0_ the baseline lifetime risk for lung cancer mortality in the age at the start of screening. The value 10 was chosen to provide a conservative estimate of the required reduction, as both the estimated radiation risk and the estimate of the reduction in lung cancer mortality are prone to uncertainties.

For the benefit–risk assessments, annual screening is assumed. Different scenarios are considered according to age and duration: for the age groups 50 to 70, 50 to 75, 55 to 75 and 60 to 75 years. In addition, screening scenarios are assumed over a period of ten years for different ages at the first screening examination.

## 4. Results

Representative organ-specific dose coefficients used for the estimation of the required organ doses are summarized in Table 2. Multiplying these coefficients by the *CTDI*_vol_ of any LDCT scan used for lung cancer screening provides representative organ doses. 

**Figure 2:**
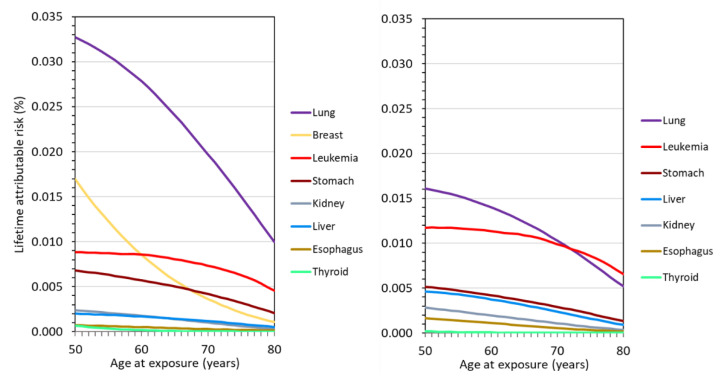
Organ-specific lifetime attributable risks, *LAR*, (cancer incidence) for women (**left**) and men (**right**) as functions of age at exposure for organ equivalent doses of 10 mSv for the German general population.

Figure 2 gives the organ-specific *LAR* for cancer incidence for organ equivalent doses of 10 mSv for the German general population separately for women and men as a function of age at exposure from 50 years on. For technical reasons (over-scanning), radiation-sensitive organs adjacent to the actual scan region, the lung, are also exposed with multi-slice CT systems. As the *LARs* for these organs contribute to the total *LAR* when considering lung cancer screening, they are included in Figure 2. The organ equivalent dose of the heart is comparable to the lung dose but is of no relevance for the radiation-related cancer risk, as cancers of the heart are extremely rare. For women and exposure age up to about 60 years, the highest radiation risks are those for lung and breast cancer, while at higher ages the radiation risks for lung cancer and leukemia dominate. It is striking that the radiation risk for lung cancer is significantly higher for women than for men.

**Figure 3:**
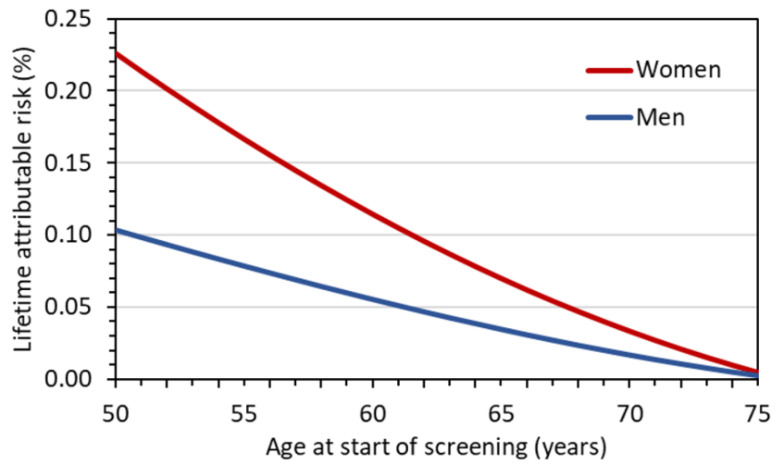
Lifetime attributable risks, *LAR*, (cancer incidence) as a function of age at first screening examination assuming annual LDCT screening up to age 75 for a representative LDCT protocol with *CTDI*_vol_ = 1 mGy.

**Figure 4:**
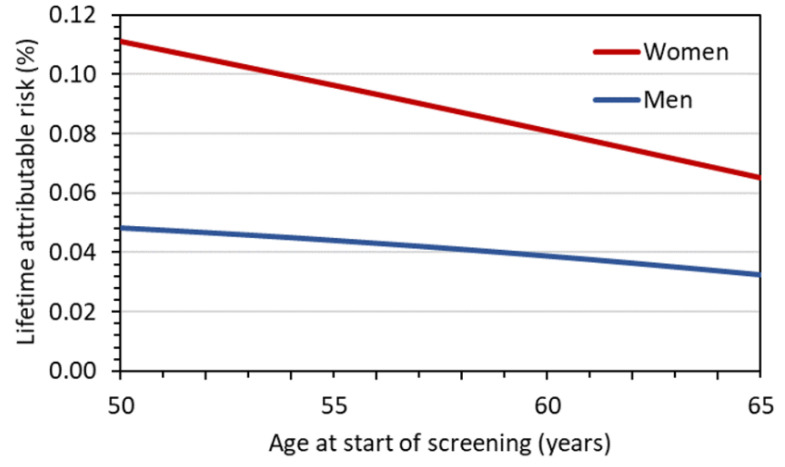
Lifetime attributable risks, *LAR*, (cancer incidence) as a function of age at first screening examination assuming annual LDCT screening over ten years for a representative LDCT protocol with *CTDI*_vol_ = 1 mGy.

Figure 3 shows for a representative LDCT protocol with a *CTDI*_vol_ of 1 mGy the *LAR* for females and males as a function of age at the start of screening, assuming annual LDCT screening until the age of 75 years. The *LAR* estimates for women are 0.23%, 0.11%, and 0.03% if screening starts at age 50, 60, and 70 years, respectively. The estimates in Figure 4 are based on the assumption that LDCT screening examinations are performed annually only over a ten-year period. In this case, *LAR* estimates for women are 0.11% and 0.08% at starting ages 50 and 60, respectively. In all screening scenarios, the risk estimates for women are slightly twice as high as those for men. The reasons for this are that (i) the radiation-related lung cancer risk according to BEIR VII is higher for women than for men and, (ii) for women the excess breast cancer risk must also be accounted for. The *LAR* values given in Figure 3 and Figure 4 are halved when LDCTs are performed biannually rather than annually.

**Figure 5:**
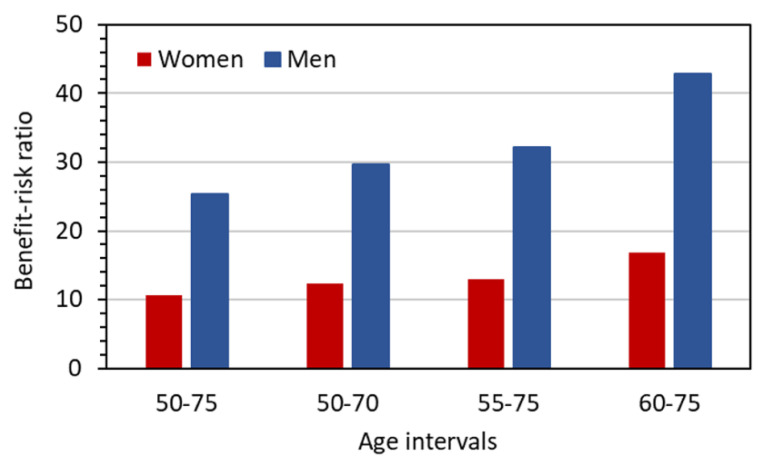
Benefit–risk ratio assuming a 20% reduction in lung cancer mortality from annual screening across the given age intervals for a representative LDCT protocol with *CTDI*_vol_ = 1 mGy.

Assuming annual screening and a 20% overall benefit for both sexes, Figure 5 gives estimates of the benefit–risk ratio for the considered screening scenarios. For men, the ratio is about 25 and above, but for females it is below 15 in three of the four screening scenarios. It should be noted that the benefit assumed to be constant for the scenarios considered was derived from screening studies in which LDCTs were performed over much shorter screening periods (8 to 10 years) and in some cases with longer intervals between LDCT examinations (up to 2.5 years). Under the assumption, which can be considered realistic, that the benefit increases with both longer screening periods and the frequency of examinations, the benefit–risk ratios given in the figure for the four scenarios are underestimated and can thus be regarded as conservative estimates. 

**Figure 6:**
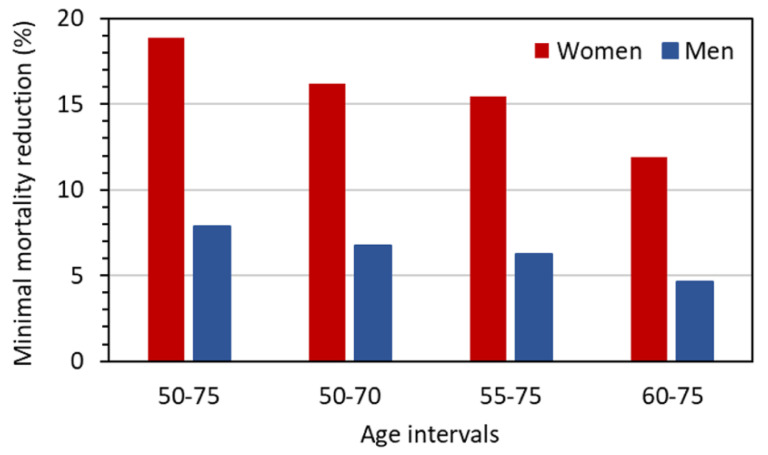
Reduction in lung cancer mortality that must be achieved to attain a benefit–risk ratio of at least 10 assuming annual screening across the given age intervals for a representative LDCT protocol with *CTDI*_vol_ = 1 mGy.

**Figure 7:**
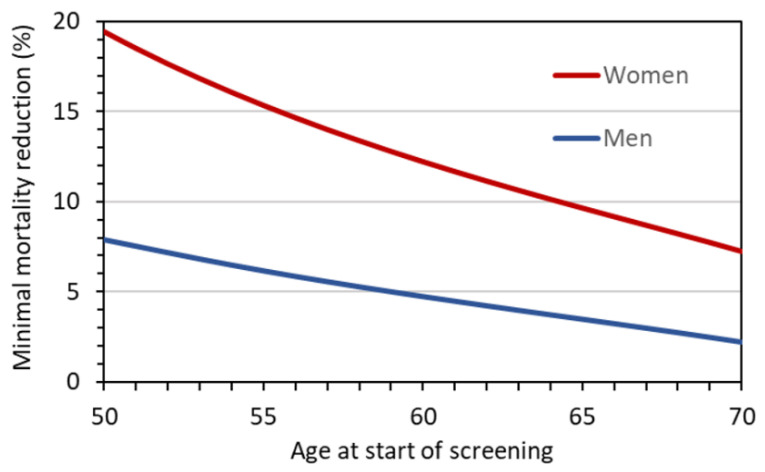
Reduction in lung cancer mortality that must be achieved to attain a benefit–risk ratio of at least 10 as a function of age at first screening examination assuming annual LDCT screening up to age 75 years for a representative LDCT protocol with *CTDI*_vol_ = 1 mGy.

The problem of uncertainty in the benefit can be addressed by considering the values for the required reduction of lung cancer mortality, *MR*_10_, by LDCT screening to achieve at least a pre-defined benefit–risk ratio for the considered screening scenarios. These *MR*_10_ values are given in Figure 6. While the values for men range between 5% and 8%, those for women are considerably higher and reach almost 20% for a screening with annual LDCT examinations from 50 to 75 years of age. Figure 7 gives *MR*_10_ as a function of age at the start of screening, assuming annual LDCT screening until the age of 75 years. For men, the minimum required reduction decreases from about 8 to 2% with increasing age at first screening examination. For women, the required reduction is 20% if screening starts at age 50 and takes values below 10% only if screening starts at age 64 or later.

## 5. Discussion

The estimated lifetime attributable cancer risks resulting from repeated LDCT screening tests were estimated for a German population of heavy smokers or ex-smokers. The values can be considered representative for a Western European population. As demonstrated, the estimated *LAR* are not negligible, especially for women if screening starts at the age of 50 years. From a radiation protection perspective, they must be taken into consideration in any decision-making, since all screening participants are affected, whereas the benefit only applies to those with screening-detected lung cancer. However, it has to be underlined that the assumptions for the above risk estimates and risk-benefit analyses are mostly conservative, for example by using a DDREF of 1 and by assuming that screening will be performed annually for the full screening period.

Moreover, it should be considered that the potential benefit for patients with a screening-detected lung cancer would be immediate, whereas a radiation-associated cancer is hypothetical and would only occur after a latency time of several years or even decades. The latency period is an important factor, especially for participants who only take part in screening at an older age.

The estimates presented in Figure 3 and Figure 4 refer to organ equivalent dose values for an LDCT protocol with *CTDI*_vol_ = 1 mGy. Since the risk is linearly dependent on *CTDI*_vol_, the estimates can easily be converted for other protocols. In a similar line of reasoning, the benefit–risk ratios given in Figure 5 can be adapted to other protocols because they are inversely proportional to the risk. Moreover, the ratios in Figure 5, based on the assumption of a 20% reduction in lung cancer mortality due to LDCT screening, can easily be transferred to screening scenarios with other values for the benefit as the benefit–risk ratio is proportional to the benefit. This is particularly useful when considering women participating in an LDCT screening, since there is evidence that the benefit for women is higher than that for men [29]. 

There is a controversial discussion in the literature about which baseline risks should be assumed for female smokers. For the estimates presented in this paper, it was assumed that the lung cancer risk for smoking women corresponds to that for smoking men. If female heavy smokers had a different (presumably higher) smoking-related lung cancer risk, this would affect the radiation risk estimates for women as well as the corresponding benefit–risk ratio. The higher weighting of the additive model in the BEIR VII approach for lung cancer implies that in this case the benefit–risk ratio would be more affected than the radiation risk.

Various publications have addressed the radiation risk of LDCT screening. Some authors (e.g., [30]) estimate the radiation risk by applying the effective dose and the nominal risk coefficients of the ICRP. The ICRP’s risk estimates are called ‘nominal’ because “they relate to the exposure of a nominal population of females and males with a typical age distribution and are computed by averaging over age groups and both sexes”. The ICRP thus explicitly states that “for the estimation of the likely consequences of an exposure of an individual or a known population, it is necessary to use specific data relating to the exposed individual” [7]. 

In the Italian COSMOS study (a non-randomized screening study of approximately 3400 men and 1800 women aged 50 years and older and up to 10 annual screening rounds), organ doses were estimated for both annual LDCT screening and recall (PET/CT) examinations in individuals with suspicious pulmonary foci [31]. Based on these data, the excess lifetime risks (incidence) for all major cancer types were determined using the original BEIR VII estimates of *LAR* for a US population in 1999. *LARs* were approximately 4 per 10,000 for men and 8 per 10,000 for women, assuming a screening start at age 50. Considering the differences between the risk analysis used by the authors and our approach (e.g., *DDREF* = 1.5 in Rampinelli et al. [31]) and different doses, the estimates are compatible. 

Radiological LDCT examinations to clarify suspicious findings during screening increase the dose and thus also the radiation risk for some participants. Becker et al. [32] give the frequency of recall LDCT for the first 5 screening rounds (T0 to T4) for the LUSI study. In round T0 about 20% of the participants had a recall LDCT after 3 or 6 months and in the following rounds on average about 3.5%. Under these assumptions, the lifetime risks after annual LDCT screening from age 50 until age 75 increase by about 4%. 

No uncertainty figures for the risk and benefit–risk estimates are provided in our study. Radiation risk estimates are subject to numerous uncertainty factors because of the inherent limitations of (radio-)epidemiological data. In addition to statistical uncertainty, the populations and exposures for which a risk estimate is to be made differ from those in radio-epidemiological studies. This means that different “educated guesses” have to be made, which are inevitably subject to uncertainties. Despite the large amount of epidemiological and experimental data on radiation risks, these data are not sufficient to give a realistic uncertainty for these guesses [5]. In a paper by Zhang et al. [33], a sensitivity analysis was performed to investigate the impact of 12 different parameters or methodological assumptions on the radiation risk estimate for solid tumors. This sensitivity analysis indicated that DDREF, age at exposure, sex, and lethality strongly influence radiation risk and that the risk transfer model also has a noticeable impact. In particular, our assumptions on the DDREF and the transfer model thus contribute to the uncertainty of our risk estimates, but they were chosen in such a way that the estimated values can be assumed to be conservative.

Radiation-associated noncancer diseases were outside the scope of our study. Nevertheless, they might be a matter of concern when it comes to an LDCT screening. An association between radiation exposure and noncancer respiratory diseases (NCRD) has been reported in the LSS of atomic bomb survivors [34]. However, the corresponding radiation-related risk was not increased in the period up to 1980, i.e., for up to 35 years after the bombings. Moreover, the majority of NCRDs were pneumonia or influenza, and it was only in this category that a significant radiation risk was found. As mentioned, the organ with a comparably high organ equivalent dose as that of the lung is the heart. Radiation-related heart diseases, however, such as ischemic heart disease, do not play a role in the low dose range. In the LSS, the lowest dose above which a significant increase in the risk of heart disease was observed is given as 0.7 Gy [35]. This is in line with the view of the ICRP, which has proposed a “practical” threshold dose of 0.5 Gy for radiation-related cardiovascular diseases that manifest late after irradiation [36].

## 6. Conclusions

Although organ equivalent doses resulting from a single LDCT scan are low, estimated lifetime attributable cancer risks resulting from an annual LDCT screening of heavy (ex-)smokers aged between 50 and 75 years cannot be neglected, especially for women. Assuming a mortality reduction of about 20% for both sexes and considering only radiation risks, this screening scenario resulted in a benefit–risk ratio of 25 for male and about 10 for female screening participants. However, the benefit–risk ratio for women may be closer to that for men, as there is evidence that the benefit of LDCT screening is larger for women than for men. The presented results can very easily be adjusted to any CT protocol and scanner via the *CTDI*_vol_ as well as to the outcome of future trials concerning the benefit of lung cancer screening. To achieve at least the benefit demonstrated in the RCTs published so far, a high level of quality along the entire screening process and an adequate evaluation of the quality of the procedures and outcomes are required for systematic screening in heavy (ex-)smokers. In addition, the participants must be informed in detail about the expected benefits and possible risks.

## Figures and Tables

**Table 1:** Differences between the BEIR VII Committee’s assessment of the *LAR* and our assessment.

BEIR VII	Modifications Compared to BEIR VII
*DDREF* = 1.5	*DDREF* = 1.0
Risk estimation based on US cancer rates and mortality tables from the 1990s	Risk estimation based on German cancer rates and mortality tables from 2016
Estimation of the *LAR* for breast cancer based on the absolute risk model	Estimation of the *LAR* for breast cancer based on both the absolute and the relative risk model with weighting *w*_abs_ = 0.7 and *w*_rel_ = 0.3

*DDREF*: Dose and dose rate effectiveness factor; *w*_abs_: weighting factor for the *LAR* based on the absolute risk model; *w*_rel_: weighting factor for the *LAR* based on the relative risk model.

**Table 2:** Representative organ-specific dose coefficients for a female or male reference person. Only those organs were considered that are located—at least in part—in the radiation field and contribute to the *LAR*.

Organ	Dose Coefficients (Mean ± Standard Deviation) (mSv/mGy)	
	Women	Men
Thyroid	2.2 ± 0.44	1.9 ± 0.46
Female breast	1.7 ± 0.11	
Lung	1.7 ± 0.10	1.7 ± 0.11
Oesophagus	1.6 ± 0.10	1.6 ± 0.12
Liver	1.0 ± 0.11	0.9 ± 0.11
Stomach	0.8 ± 0.13	0.7 ± 0.14
Red bone marrow	0.5 ± 0.03	0.5 ± 0.04
Kidney	0.4 ± 0.16	0.4 ± 0.15
